# Supplementary material for: Modeling glioblastoma heterogeneity as a dynamic network of cell states
Source: Mol Syst Biol. 2021 Sep 16;17(9):e10105. doi: 10.15252/msb.202010105 (PMC8444284; doi:10.15252/msb.202010105)
Supplement: Supplementary file 5 — Source Data for Figure 3 [file MSB-17-e10105-s001.zip › Figure3A_sourcedata/GSEA_3065/hallmarks_state6.GseaPreranked.1623416308702/HALLMARK_P53_PATHWAY.html]

Details for gene set HALLMARK\_P53\_PATHWAY[GSEA]

|  || Dataset | state6 |
| Phenotype | NoPhenotypeAvailable |
| Upregulated in class | na\_pos |
| GeneSet | HALLMARK\_P53\_PATHWAY |
| Enrichment Score (ES) | 0.5727335 |
| Normalized Enrichment Score (NES) | 2.5267193 |
| Nominal p-value | 0.0 |
| FDR q-value | 0.0 |
| FWER p-Value | 0.0 |
Table: GSEA Results Summary

  

Fig 1: Enrichment plot: HALLMARK\_P53\_PATHWAY      
 Profile of the Running ES Score & Positions of GeneSet Members on the Rank Ordered List

  

| PROBE | GENE SYMBOL | GENE\_TITLE | RANK IN GENE LIST | RANK METRIC SCORE | RUNNING ES | CORE ENRICHMENT || 1 | HMOX1 |  |  | 0 | 0.545 | 0.0430 | Yes |
| 2 | CDKN1A |  |  | 1 | 0.477 | 0.0805 | Yes |
| 3 | GADD45A |  |  | 2 | 0.419 | 0.1135 | Yes |
| 4 | BTG1 |  |  | 4 | 0.348 | 0.1408 | Yes |
| 5 | PHLDA3 |  |  | 29 | 0.272 | 0.1594 | Yes |
| 6 | CCND2 |  |  | 32 | 0.269 | 0.1804 | Yes |
| 7 | RPL18 |  |  | 36 | 0.256 | 0.2002 | Yes |
| 8 | ZMAT3 |  |  | 40 | 0.252 | 0.2197 | Yes |
| 9 | PLK2 |  |  | 43 | 0.249 | 0.2391 | Yes |
| 10 | FDXR |  |  | 56 | 0.241 | 0.2567 | Yes |
| 11 | RACK1 |  |  | 57 | 0.240 | 0.2756 | Yes |
| 12 | BTG2 |  |  | 63 | 0.234 | 0.2935 | Yes |
| 13 | BAX |  |  | 65 | 0.233 | 0.3117 | Yes |
| 14 | RPS27L |  |  | 91 | 0.218 | 0.3261 | Yes |
| 15 | TP53 |  |  | 116 | 0.205 | 0.3394 | Yes |
| 16 | AEN |  |  | 128 | 0.200 | 0.3539 | Yes |
| 17 | MDM2 |  |  | 155 | 0.189 | 0.3659 | Yes |
| 18 | RPS12 |  |  | 164 | 0.187 | 0.3797 | Yes |
| 19 | RPL36 |  |  | 166 | 0.187 | 0.3942 | Yes |
| 20 | TXNIP |  |  | 176 | 0.183 | 0.4076 | Yes |
| 21 | DDIT3 |  |  | 205 | 0.175 | 0.4182 | Yes |
| 22 | TRIAP1 |  |  | 214 | 0.170 | 0.4307 | Yes |
| 23 | RGS16 |  |  | 239 | 0.164 | 0.4408 | Yes |
| 24 | TRAF4 |  |  | 244 | 0.163 | 0.4532 | Yes |
| 25 | HRAS |  |  | 245 | 0.163 | 0.4660 | Yes |
| 26 | ZFP36L1 |  |  | 266 | 0.159 | 0.4762 | Yes |
| 27 | HINT1 |  |  | 274 | 0.156 | 0.4877 | Yes |
| 28 | RAP2B |  |  | 280 | 0.154 | 0.4993 | Yes |
| 29 | EI24 |  |  | 317 | 0.149 | 0.5069 | Yes |
| 30 | CCNG1 |  |  | 319 | 0.148 | 0.5184 | Yes |
| 31 | DDIT4 |  |  | 357 | 0.142 | 0.5254 | Yes |
| 32 | PPM1D |  |  | 386 | 0.139 | 0.5331 | Yes |
| 33 | TSC22D1 |  |  | 398 | 0.136 | 0.5425 | Yes |
| 34 | CYFIP2 |  |  | 436 | 0.130 | 0.5485 | Yes |
| 35 | ATF3 |  |  | 494 | 0.124 | 0.5517 | Yes |
| 36 | MXD4 |  |  | 495 | 0.124 | 0.5615 | Yes |
| 37 | FBXW7 |  |  | 498 | 0.124 | 0.5710 | Yes |
| 38 | SDC1 |  |  | 698 | 0.106 | 0.5564 | Yes |
| 39 | SAT1 |  |  | 744 | 0.102 | 0.5592 | Yes |
| 40 | TOB1 |  |  | 746 | 0.102 | 0.5672 | Yes |
| 41 | NINJ1 |  |  | 819 | 0.097 | 0.5665 | Yes |
| 42 | SESN1 |  |  | 965 | 0.087 | 0.5567 | Yes |
| 43 | PPP1R15A |  |  | 968 | 0.087 | 0.5633 | Yes |
| 44 | IP6K2 |  |  | 1001 | 0.086 | 0.5663 | Yes |
| 45 | HSPA4L |  |  | 1005 | 0.085 | 0.5727 | Yes |
| 46 | PRMT2 |  |  | 1174 | 0.077 | 0.5594 | No |
| 47 | ISCU |  |  | 1241 | 0.074 | 0.5576 | No |
| 48 | APAF1 |  |  | 1397 | 0.068 | 0.5450 | No |
| 49 | TAX1BP3 |  |  | 1400 | 0.067 | 0.5501 | No |
| 50 | HEXIM1 |  |  | 1412 | 0.067 | 0.5541 | No |
| 51 | ABAT |  |  | 1484 | 0.065 | 0.5510 | No |
| 52 | NDRG1 |  |  | 1488 | 0.065 | 0.5558 | No |
| 53 | BAIAP2 |  |  | 1539 | 0.062 | 0.5549 | No |
| 54 | STEAP3 |  |  | 1674 | 0.058 | 0.5440 | No |
| 55 | WRAP73 |  |  | 1678 | 0.058 | 0.5482 | No |
| 56 | KIF13B |  |  | 1879 | 0.051 | 0.5292 | No |
| 57 | DDB2 |  |  | 2045 | 0.046 | 0.5138 | No |
| 58 | BAK1 |  |  | 2122 | 0.044 | 0.5085 | No |
| 59 | FOXO3 |  |  | 2178 | 0.042 | 0.5055 | No |
| 60 | RXRA |  |  | 2249 | 0.040 | 0.5006 | No |
| 61 | DCXR |  |  | 2396 | 0.036 | 0.4866 | No |
| 62 | ABCC5 |  |  | 2430 | 0.035 | 0.4855 | No |
| 63 | ANKRA2 |  |  | 2593 | 0.031 | 0.4693 | No |
| 64 | UPP1 |  |  | 2662 | 0.030 | 0.4638 | No |
| 65 | PITPNC1 |  |  | 2701 | 0.029 | 0.4616 | No |
| 66 | CCP110 |  |  | 2781 | 0.027 | 0.4547 | No |
| 67 | SEC61A1 |  |  | 2806 | 0.026 | 0.4540 | No |
| 68 | RALGDS |  |  | 2917 | 0.024 | 0.4432 | No |
| 69 | SERTAD3 |  |  | 3062 | 0.021 | 0.4282 | No |
| 70 | POM121 |  |  | 3071 | 0.021 | 0.4289 | No |
| 71 | MAPKAPK3 |  |  | 3159 | 0.019 | 0.4203 | No |
| 72 | FUCA1 |  |  | 3169 | 0.018 | 0.4207 | No |
| 73 | PLK3 |  |  | 3197 | 0.018 | 0.4190 | No |
| 74 | TRAFD1 |  |  | 3306 | 0.015 | 0.4077 | No |
| 75 | WWP1 |  |  | 3347 | 0.014 | 0.4043 | No |
| 76 | NOTCH1 |  |  | 3459 | 0.012 | 0.3924 | No |
| 77 | CSRNP2 |  |  | 3510 | 0.011 | 0.3875 | No |
| 78 | CDKN2AIP |  |  | 3581 | 0.010 | 0.3802 | No |
| 79 | RAD51C |  |  | 3629 | 0.009 | 0.3754 | No |
| 80 | VWA5A |  |  | 3735 | 0.006 | 0.3638 | No |
| 81 | RAB40C |  |  | 3806 | 0.005 | 0.3560 | No |
| 82 | MKNK2 |  |  | 3866 | 0.003 | 0.3495 | No |
| 83 | FAM162A |  |  | 3867 | 0.003 | 0.3498 | No |
| 84 | EPHA2 |  |  | 3952 | 0.001 | 0.3402 | No |
| 85 | BLCAP |  |  | 3962 | 0.001 | 0.3392 | No |
| 86 | PERP |  |  | 3975 | 0.001 | 0.3379 | No |
| 87 | CCND3 |  |  | 4016 | 0.000 | 0.3334 | No |
| 88 | ZNF365 |  |  | 4042 | -0.000 | 0.3305 | No |
| 89 | CGRRF1 |  |  | 4110 | -0.002 | 0.3229 | No |
| 90 | ELP1 |  |  | 4194 | -0.003 | 0.3135 | No |
| 91 | RCHY1 |  |  | 4221 | -0.004 | 0.3108 | No |
| 92 | PRKAB1 |  |  | 4288 | -0.005 | 0.3036 | No |
| 93 | PMM1 |  |  | 4411 | -0.007 | 0.2900 | No |
| 94 | FOS |  |  | 4536 | -0.010 | 0.2765 | No |
| 95 | LDHB |  |  | 4572 | -0.010 | 0.2732 | No |
| 96 | RRP8 |  |  | 4808 | -0.014 | 0.2473 | No |
| 97 | PTPN14 |  |  | 4849 | -0.015 | 0.2439 | No |
| 98 | HDAC3 |  |  | 4856 | -0.016 | 0.2444 | No |
| 99 | SLC35D1 |  |  | 4889 | -0.016 | 0.2420 | No |
| 100 | DRAM1 |  |  | 5008 | -0.018 | 0.2298 | No |
| 101 | STOM |  |  | 5231 | -0.023 | 0.2060 | No |
| 102 | S100A10 |  |  | 5285 | -0.024 | 0.2018 | No |
| 103 | SLC7A11 |  |  | 5291 | -0.024 | 0.2031 | No |
| 104 | PIDD1 |  |  | 5424 | -0.027 | 0.1900 | No |
| 105 | PDGFA |  |  | 5426 | -0.027 | 0.1920 | No |
| 106 | PTPRE |  |  | 5684 | -0.032 | 0.1648 | No |
| 107 | TM7SF3 |  |  | 5744 | -0.033 | 0.1607 | No |
| 108 | XPC |  |  | 5756 | -0.034 | 0.1620 | No |
| 109 | F2R |  |  | 5881 | -0.036 | 0.1505 | No |
| 110 | NOL8 |  |  | 6069 | -0.040 | 0.1321 | No |
| 111 | ITGB4 |  |  | 6517 | -0.050 | 0.0844 | No |
| 112 | PLXNB2 |  |  | 6565 | -0.052 | 0.0831 | No |
| 113 | CD82 |  |  | 6649 | -0.054 | 0.0778 | No |
| 114 | IRAK1 |  |  | 6677 | -0.055 | 0.0790 | No |
| 115 | TGFB1 |  |  | 6712 | -0.056 | 0.0795 | No |
| 116 | EPHX1 |  |  | 7008 | -0.065 | 0.0506 | No |
| 117 | SLC3A2 |  |  | 7118 | -0.068 | 0.0434 | No |
| 118 | CTSD |  |  | 7147 | -0.069 | 0.0456 | No |
| 119 | SP1 |  |  | 7289 | -0.074 | 0.0352 | No |
| 120 | BMP2 |  |  | 7299 | -0.074 | 0.0400 | No |
| 121 | CTSF |  |  | 7309 | -0.075 | 0.0448 | No |
| 122 | IER5 |  |  | 7383 | -0.077 | 0.0425 | No |
| 123 | JUN |  |  | 7513 | -0.082 | 0.0341 | No |
| 124 | PCNA |  |  | 8061 | -0.111 | -0.0203 | No |
| 125 | DNTTIP2 |  |  | 8170 | -0.119 | -0.0234 | No |
| 126 | RAD9A |  |  | 8184 | -0.120 | -0.0155 | No |
| 127 | CCNK |  |  | 8358 | -0.141 | -0.0244 | No |
| 128 | TM4SF1 |  |  | 8392 | -0.146 | -0.0167 | No |
| 129 | TAP1 |  |  | 8393 | -0.146 | -0.0052 | No |
| 130 | TPRKB |  |  | 8438 | -0.151 | 0.0016 | No |
| 131 | CD81 |  |  | 8625 | -0.201 | -0.0041 | No |
| 132 | APP |  |  | 8758 | -0.300 | 0.0044 | No |
Table: GSEA details [plain text format]

  

Fig 2: HALLMARK\_P53\_PATHWAY: Random ES distribution      
 Gene set null distribution of ES for **HALLMARK\_P53\_PATHWAY**
